# Oral health status and preventive determinants in children with type 1 diabetes: a cross-sectional comparative study

**DOI:** 10.3389/fdmed.2026.1868866

**Published:** 2026-06-24

**Authors:** Fatima Ezzahra Zidane, Soukaina Rouijel, Rachid Fawzi

**Affiliations:** 1 International Faculty of Dental Medicine, Health Sciences Research Center (CReSS), College of Health Sciences, International University of Rabat, Sala Al Jadida, Morocco; 2 Department of Pediatric Dentistry, Marrakesh Private Hospital, Private University of Marrakech, Marrakech, Morocco

**Keywords:** dental caries, gingivitis, oral hygiene, pediatric oral health, preventive dentistry, type 1 diabetes

## Abstract

**Background:**

Type 1 diabetes mellitus (T1DM) is a chronic metabolic disorder that may adversely affect oral health in children. However, the contribution of preventive determinants to oral health outcomes in this population remains insufficiently explored.

**Objective:**

To compare oral health status and preventive determinants between children with T1DM and non-diabetic peers and to investigate their associations with oral health outcomes.

**Methods:**

A cross-sectional study was conducted among 100 children with T1DM and 100 age- and sex-matched non-diabetic controls (3–17 years). Data on oral hygiene practices, dietary habits, and dental attendance were collected using structured questionnaires. Clinical examinations assessed dental caries (DMFT/dmft), plaque index (PI), gingival index (GI), and calculus deposition. Statistical analyses included chi-square tests, Student's t-tests, and ANOVA.

**Results:**

The mean age of participants was 9.6 years. No significant difference in DMFT/dmft was observed between groups (*p* = 0.60). Children with T1DM exhibited higher plaque index values (*p* = 0.05), a greater prevalence of calculus deposition (*p* = 0.001), and more severe gingival inflammation (*p* = 0.022). Poor glycemic control was significantly associated with gingival inflammation (*p* = 0.043). Incorrect toothbrushing technique was more frequent among children with T1DM (*p* < 0.001), and dietary behavior was associated with DMFT/dmft in this group (*p* = 0.02).

**Conclusion:**

Children with T1DM exhibited poorer periodontal and oral hygiene status than non-diabetic peers despite similar caries experience. Oral hygiene practices and dietary behaviors were significantly associated with oral health outcomes. These findings support the integration of targeted oral health promotion strategies into routine pediatric diabetes care.

## 1 Introduction

Type 1 diabetes mellitus (T1DM) is a chronic metabolic disorder characterized by autoimmune destruction of pancreatic β-cells, resulting in insulin deficiency and persistent hyperglycemia (1). It predominantly affects children and adolescents and accounts for approximately 5%–10% of all diabetes cases worldwide (2). Its incidence has steadily increased over recent decades, representing a growing public health concern, particularly in low- and middle-income countries (3). In Morocco, the prevalence of diabetes has risen markedly, reflecting an increasing burden in both adult and pediatric populations.

Beyond its systemic implications, T1DM has been associated with various oral health alterations. Children with T1DM are more prone to dental plaque accumulation, gingival inflammation, periodontal disease, and changes in oral tissues (4, 5). These manifestations may be explained by the effects of chronic hyperglycemia on salivary flow and composition, as well as on the host immune response (6). Reduced salivary flow and increased glucose concentration in saliva may promote bacterial growth and contribute to dental caries and periodontal inflammation (7, 25), while immune dysfunction may increase susceptibility to infections and impair healing processes (8).

Despite growing interest in this topic, evidence regarding the relationship between T1DM and oral health remains inconsistent, particularly concerning dental caries. While some studies report a higher prevalence of caries in children with T1DM, others show no significant differences compared to non-diabetic peers (9, 10). In contrast, there is stronger evidence supporting an increased susceptibility to gingival inflammation and periodontal disease, especially in cases of poor glycemic control (11, 12). These discrepancies suggest that oral health outcomes are linked not only by biological mechanisms but also by behavioral and environmental factors.

Preventive determinants—including oral hygiene practices, dietary habits, and dental care utilization—play a central role in maintaining oral health and preventing disease progression (5, 13, 19). These factors are fundamental to caries prevention strategies in pediatric populations, as they are associated with cariogenic biofilm accumulation and dietary sugar exposure (14). Regular toothbrushing contributes to effective plaque control, while appropriate dietary habits—particularly reduced frequency of cariogenic snacking—help lower caries risk. Regular dental attendance further supports early detection and timely management of oral diseases. In children with T1DM, these preventive behaviors are especially critical, as they may mitigate the adverse oral effects associated with the disease.

However, preventive determinants remain insufficiently explored, particularly in studies integrating both behavioral and clinical dimensions. Most existing research has primarily focused on clinical oral health indicators, with limited attention to modifiable behavioral factors such as oral hygiene habits, dietary patterns, and dental attendance. In our previous study conducted among Moroccan children with T1DM, altered oral health status was reported; however, the role of preventive determinants in shaping these outcomes was not specifically investigated (15). Moreover, comparative studies evaluating both preventive behaviors and oral health outcomes in diabetic and non-diabetic pediatric populations remain scarce, especially in developing countries such as Morocco.

Therefore, this study aims to: (1) describe the oral health status of children with T1DM, (2) compare these findings with those of non-diabetic children, and (3) explore the relationships between preventive determinants—including oral hygiene practices, dietary habits, and dental care utilization—and oral health outcomes. By integrating clinical and behavioral dimensions, this study seeks to provide a comprehensive understanding of oral health in children with T1DM and to inform targeted and evidence-based preventive strategies, particularly within the framework of pediatric caries prevention and integrated diabetes care.

## 2 Materials and methods

### 2.1 Study population

This descriptive cross-sectional study included 100 children with type 1 diabetes (T1DM) recruited from the Young Diabetic House in Rabat, and 100 healthy children (controls) attending public schools and participating in the national dental prevention program. Controls were frequency matched to children with T1DM according to age group and sex distribution.

A convenience sampling method was employed, and participants were recruited consecutively over a six-month period (December to June). A convenience sampling approach was selected because of the limited availability of pediatric patients with T1DM and the practical constraints associated with recruiting this specific population. To reduce selection bias, all eligible children attending the recruitment centers during the study period were consecutively invited to participate.

Inclusion criteria were: children aged 3–17 years; written informed consent obtained from parents or legal guardians; for the T1DM group, a confirmed diagnosis of type 1 diabetes mellitus established by a pediatric endocrinologist according to standard clinical and laboratory criteria and documented in the patient's medical record. For the control group, children in good general health without systemic conditions affecting oral health.

Children with other systemic diseases, those undergoing orthodontic treatment, or those who had received antibiotic therapy within the previous three months were excluded.

### 2.2 Conduct of the investigation

The study protocol was approved by the Institutional Review Board (CUMD/FIMD 003/20/24/Approval/20/24). Written informed consent was obtained from parents or legal guardians prior to participation.

All procedures were conducted in accordance with the Declaration of Helsinki. Participant confidentiality and anonymity were strictly maintained throughout the study.

### 2.3 Data collection and variables studied

Data were collected using two structured questionnaires: one for children with T1DM and one for non-diabetic children. The questionnaires were identical except for diabetes-specific variables included only for the T1DM group.

The instrument was adapted from a previously developed and validated questionnaire used in our earlier study (15), ensuring consistency and comparability.

#### 2.3.1 Questionnaire data

The following variables were collected:

- **Sociodemographic variables:**
  age, sex, place of birth, educational level of the child (preschool, primary, secondary), and parental education level (none, primary, secondary, university)
- **General health variables:**
  medical and surgical history, allergies; and for the T1DM group: age at diagnosis, duration of diabetes, number of insulin injections per day, and glycated hemoglobin (HbA1c), categorized as:
  <7.5% (good control), 7.5%–9.5% (moderate control), and >9.5% (poor control)
- **Oral hygiene variables:**
  age at initiation of toothbrushing, frequency, and duration of brushing
- **Oral health–related variables:**
  history of dental visits, age at first dental consultation, date and reason for last visit, type of dental care received, barriers to dental attendance, and parental awareness of the relationship between oral health and diabetes
- **Dietary habits:**
  dietary balance, snacking behavior (frequency and type of snacks), and, for the T1DM group, changes in eating habits following diabetes diagnosis

Dietary habits were assessed using a structured questionnaire administered to participants and/or their parents. Information was collected regarding meal frequency, number of snacks, and the usual composition of the diet. A diet was considered balanced when it included an adequate intake of proteins, lipids, and carbohydrates and followed the dietary recommendations routinely provided in the pediatric diabetes clinic, consisting of three main meals per day and two scheduled snacks (mid-morning and mid-afternoon).

#### 2.3.2 Clinical examination

All clinical examinations were performed by a single trained and calibrated examiner under standardized conditions using sterile dental instruments and adequate lighting.

Prior to data collection, calibration was performed on 10 children under the supervision of experienced clinicians. Clinical findings were reviewed and validated before initiation of the main study. Intra-examiner agreement was assessed and showed a Kappa coefficient greater than 0.80, indicating substantial agreement.

The following indices were recorded:

- Periodontal parameters:
  Plaque Index (Silness and Löe) and Gingival Index (Löe and Silness) (30).
  Plaque accumulation was assessed using the Silness and Löe Plaque Index, which evaluates the thickness of plaque deposits at the gingival margin. Gingival health was assessed using the Löe and Silness Gingival Index based on the presence of gingival inflammation, including changes in color, edema, and bleeding on gentle probing.
- Dental caries:
  DMFT/dmft index for permanent and primary dentition, according to WHO criteria
- Other oral conditions:
  presence of calculus, oral mucosal conditions, and molar incisor hypomineralization (MIH)

#### 2.3.3 Statistical analysis

Statistical analysis was performed using SPSS software (version 20.0; IBM Corp., Armonk, NY, USA).

Normality of quantitative variables was assessed using the Shapiro–Wilk test.

Quantitative variables were expressed as mean ± standard deviation, and qualitative variables as frequencies and percentages.

Descriptive analysis was conducted to characterize the study population. Comparative analyses between children with T1DM and controls were performed using:

- Chi-square test for categorical variables
- Student’s t-test for comparison of two independent groups
- One-way ANOVA for comparisons involving more than two groups

Associations between preventive determinants and oral health outcomes were further explored.

A *p*-value < 0.05 was considered statistically significant. All tests were two-tailed.

## 3 Results

All participants met the predefined inclusion criteria. The non-diabetic group consisted of children and adolescents in good general health, with no medical conditions or treatments likely to affect oral health. The overall mean age of participants was 9.6 years. For analytical purposes, participants were stratified into three age groups: 3–5 years, >5–12 years, and >12 years, corresponding to primary, mixed, and permanent dentition stages, respectively.

### 3.1 Characteristics of the study population

A total of 200 children were included, comprising 100 children with type 1 diabetes (T1DM) and 100 non-diabetic controls. The T1DM group included 53% boys and 47% girls, while the control group included 44% boys and 56% girls. The mean age was 9.83 ± 2.73 years in the T1DM group and 9.39 ± 2.57 years in controls. The majority of participants in both groups belonged to the 6–12-year age category (76%).

As shown in Table 1, the two groups were comparable in terms of age and sex distribution.

**Table 1:** Characteristics of the study population. Values are presented as mean ± standard deviation (SD) for continuous variables and as number (percentage) for categorical variables.

Variable	T1DM (*n* = 100)	Non-diabetic (*n* = 100)
Male sex	53 (53%)	44 (44%)
Female sex	47 (47%)	56 (56%)
Mean age (years)	9.83 ± 2.73	9.39 ± 2.57
Age group 3–5 years	2 (2%)	2 (2%)
Age group >5–12 years	76 (76%)	76 (76%)
Age group >12 years	22 (22%)	22 (22%)

### 3.2 Oral health status

Dental caries were highly prevalent in both groups. The mean DMFT/dmft index was slightly higher in children with T1DM (6.1 ± 3.26) compared to controls (5.85 ± 3.58), with no statistically significant difference (*p* = 0.6).

The mean plaque index was higher in the T1DM group (0.97 ± 0.28) compared to controls (0.45 ± 0.30) (*p* = 0.05).

No statistically significant difference was observed in mean gingival index values between groups (0.95 ± 0.34 in the T1DM group versus 0.98 ± 0.22 in controls; *p* = 0.47).

Distribution of gingival inflammation severity differed significantly (*p* = 0.022), with more moderate and severe forms observed among children with T1DM.

A significantly higher prevalence of calculus deposition was observed in the T1DM group (40%) compared to controls (18%) (*p* = 0.001).

Detailed comparisons are presented in Table 2.

**Table 2:** Comparison of oral health status between children with type 1 diabetes and controls.

Variable	T1DM (*n* = 100)	Non-diabetic (*n* = 100)	*p*-value
DMFT/dmft (mean ± SD)	6.1 ± 3.26	5.85 ± 3.58	0.6
Plaque Index (PI)	0.97 ± 0.28	0.45 ± 0.30	0.05
Gingival Index (GI)	0.95 ± 0.34	0.98 ± 0.22	0.47
Calculus present	40 (40%)	18 (18%)	0.001
Gingival inflammation severity			0.022
Mild	45 (45%)	59 (59%)	
Moderate	37 (37%)	25 (25%)	
Severe	2 (2%)	0 (0%)	

Values are presented as mean ± SD or number (percentage). Student's t-test was used for continuous variables and the chi-square test for categorical variables. Gingival inflammation categories were compared using the chi-square test.

Percentages for gingival inflammation severity were calculated among participants presenting gingival inflammation (*n* = 84 in each group). Mean Gingival Index values were analyzed as a continuous variable, whereas gingival inflammation severity was analyzed as a categorical variable.

### 3.3 Association with diabetes-related factors

Poor glycemic control was significantly associated with gingival inflammation (*p* = 0.043). Gingival inflammation was present in 68.2% of children with controlled diabetes, compared to 88.5% among those with uncontrolled diabetes.

No statistically significant association was observed between diabetes duration and oral health indices, including DMFT/dmft, plaque index, and gingival index (*p* > 0.05).

These findings are summarized in Table 3.

**Table 3:** Association between glycemic control and gingival inflammation.

Variable	Gingival inflammation present	Gingival inflammation absent	*p*-value
Glycemic control			0.043
Controlled	15 (68.2%)	7 (31.8%)	
Uncontrolled	69 (88.5%)	9 (11.5%)	

Values are presented as number (percentage). Associations were assessed using the chi-square test.

### 3.4 Preventive determinants and oral health outcomes

Oral hygiene practices are presented in Table 4. No significant differences were observed between groups in terms of toothbrushing frequency (*p* = 0.7) or brushing twice daily (*p* = 0.14). However, correct toothbrushing technique was significantly less frequent among children with T1DM (10.9%) compared to controls (38.8%) (*p* < 0.001).

**Table 4:** Oral hygiene practices among children with type 1 diabetes and controls.

Variable	T1DM (*n* = 100)	Non-diabetic (*n* = 100)	*p*-value
Toothbrushing			0.7
Yes	82 (82%)	85 (85%)	
No	18 (18%)	15 (15%)	
Brushing twice daily	37 (37%)	31 (31%)	0.14
Brushing technique			<0.001
Correct	9 (10.9%)	33 (38.8%)	
Incorrect	73 (89.1%)	52 (61.2%)	

Values are presented as number (percentage). Comparisons between groups were performed using the chi-square test.

Percentages for toothbrushing technique were calculated among participants who reported toothbrushing (*n* = 82 in the T1DM group and *n* = 85 in the control group).

Dental attendance patterns are shown in Table 5. Although no statistically significant difference was found (*p* = 0.55), children with T1DM tended to report less frequent dental visits, with a higher proportion indicating their last visit occurred more than one year prior.

**Table 5:** Dental attendance patterns among children with type 1 diabetes and controls.

Variable	T1DM (*n* = 100)	Non-diabetic (*n* = 100)	*p*-value
Previous dental visit			0.55
Yes	61 (61%)	65 (65%)	
No previous visit	39 (39%)	35 (35%)	
Last visit <1 year	15 (15%)	39 (39%)	
Last visit >1 year	44 (44%)	25 (25%)	

Values are presented as number (percentage). Comparisons between groups were performed using the chi-square test.

Percentages for the timing of the last dental visit were calculated using the total number of participants in each group as the denominator (*n* = 100).

Dietary habits are presented in Table 6. A balanced diet was significantly more frequent among controls (96%) compared to children with T1DM (63%) (*p* < 0.001). Snacking behavior also differed significantly between groups (*p* = 0.01).

**Table 6:** Dietary habits among children with type 1 diabetes and controls.

Variable	T1DM (*n* = 100)	Non-diabetic (*n* = 100)	*p*-value
Balanced diet			<0.001
Yes	63 (63%)	96 (96%)	
No	37 (37%)	4 (4%)	
Snacking			0.01
Yes	73 (73%)	87 (87%)	
No	27 (27%)	13 (13%)	

Values are presented as number (percentage). Comparisons between groups were performed using the chi-square test.

The association between dietary habits and oral health outcomes is detailed in Table 7. In the T1DM group, a balanced diet was significantly associated with lower DMFT values (*p* = 0.02), whereas no significant association was observed with plaque index (*p* > 0.05).

**Table 7:** Comparison of oral health outcomes between children with T1DM and non-diabetic controls according to dietary habits.

Variable	T1DM	Non-diabetic	*p*-value
DMFT/dmft (balanced diet)	5.55 ± 2.84	7.02 ± 3.73	0.02
DMFT/dmft (unbalanced diet)	5.87 ± 3.59	7.5 ± 1.91	0.37
Plaque index (balanced diet)	0.95 ± 0.25	1 ± 0.32	0.46
Plaque index (unbalanced diet)	1.05 ± 0.30	1.1 ± 0.37	0.7

Values are presented as mean ± SD. Student's t-test was used to compare mean values between children with T1DM and non-diabetic controls within each dietary category.

## 4 Discussion

This study investigated oral health status and preventive determinants in children with type 1 diabetes mellitus (T1DM) compared with non-diabetic peers. The findings indicate that children with T1DM exhibit poorer periodontal and oral hygiene conditions, particularly characterized by increased plaque accumulation, higher prevalence of calculus deposition, and greater severity of gingival inflammation. In contrast, no significant difference in dental caries experience was observed between the two groups. Preventive determinants, particularly oral hygiene quality and dietary behaviors, were also found to be associated with oral health outcomes.

One of the main findings of this study is the significantly higher level of calculus deposition and greater severity of gingival inflammation in children with T1DM. These results support the well-established relationship between diabetes and periodontal conditions, which is primarily mediated by chronic hyperglycemia, altered immune response, and increased inflammatory burden (7, 8, 16).

The observed association between poor glycemic control and gingival inflammation further emphasizes the role of metabolic regulation as a key determinant of periodontal health. This finding suggests that gingival inflammation in children with T1DM may reflect not only local factors such as plaque accumulation but also systemic metabolic imbalance (4–6, 8, 12). Similar findings have been consistently reported in pediatric populations, where gingivitis is more frequent and severe in children with diabetes (11, 12, 28).

These results are consistent with our previous study conducted among Moroccan children with T1DM, which reported compromised oral health status (15). However, the present study extends these findings by incorporating preventive and behavioral determinants, providing a more comprehensive and clinically relevant understanding of oral health in this population.

In contrast, no statistically significant difference in DMFT/dmft index was observed between diabetic and non-diabetic children. This finding aligns with several studies reporting comparable caries experience in children with T1DM (9, 10, 18). Possible explanations include dietary restrictions, closer medical supervision, and increased parental awareness, which may contribute to controlling cariogenic exposure despite the presence of systemic disease.

Several biological mechanisms may explain the potential association between T1DM and dental caries in children. Chronic hyperglycemia may alter salivary composition and reduce salivary flow, resulting in decreased buffering capacity, impaired oral clearance, and reduced remineralization potential. Increased salivary glucose concentrations may promote the growth of cariogenic microorganisms, particularly acidogenic and aciduric bacterial species, while diabetes-related immune dysfunction may further modify the oral microbial environment and increase susceptibility to oral disease. In addition, fluctuations in metabolic control may influence salivary pH and oral ecological balance, potentially favoring cariogenic processes (26). However, these biological factors may be counterbalanced by dietary supervision, reduced consumption of refined sugars, regular medical follow-up, and increased parental awareness, which are commonly observed among children with T1DM. These protective factors may contribute to the absence of significant differences in caries experience between diabetic and non-diabetic children in the present study (27). Importantly, although caries experience did not differ between groups, the observed behavioral differences—particularly in oral hygiene quality and dietary habits—are well-established determinants of caries risk and may have long-term implications for disease development.

The significant association between poor glycemic control and gingival inflammation further supports the concept that metabolic control plays a central role in periodontal health (29). This relationship is biologically plausible, as hyperglycemia affects host defense mechanisms, including impaired neutrophil function, increased production of pro-inflammatory cytokines, and vascular alterations (4, 6, 17). Additionally, the bidirectional relationship between diabetes and periodontal disease suggests that periodontal inflammation may, in turn, negatively affect glycemic control (6, 19, 21).

Although plaque index values were higher in children with T1DM, the borderline statistical significance suggests that differences in oral hygiene quantity alone may not fully explain the observed periodontal alterations. Instead, qualitative aspects of oral hygiene practices appear to be more critical. This interpretation is supported by the finding that correct toothbrushing technique was significantly less frequent among diabetic children, indicating that ineffective plaque removal may contribute to persistent gingival inflammation despite similar brushing frequency (28).

Preventive determinants emerged as key factors associated with oral health outcomes (22). While toothbrushing frequency did not differ significantly between groups, brushing technique was markedly poorer among children with T1DM, emphasizing that the quality of oral hygiene practices may be more important than frequency alone.

Dietary habits also differed significantly between groups. A balanced diet was less frequently reported among children with T1DM, and dietary behavior was significantly associated with DMFT/dmft in this group. Diet is a well-established factor associated with oral health, particularly in relation to dental caries, and these findings suggest that dietary patterns may be associated with oral health outcomes in children with T1DM (14, 20, 24).

Although no statistically significant differences were observed in dental attendance, fewer children with T1DM reported recent dental visits. This trend may reflect barriers to accessing dental care or a prioritization of medical over dental follow-up in chronic disease management. Delayed dental consultations may be associated with poorer oral health outcomes and highlight the need for better integration of oral health services into routine diabetes care (13, 23).

Taken together, these findings highlight the relationship between metabolic control and behavioral factors, suggesting that oral health outcomes in children with T1DM are associated with both disease-related and behavioral characteristics.

From a translational and clinical perspective, these results underscore the need to integrate oral health promotion into comprehensive pediatric diabetes care (24). Preventive strategies should include structured education on effective toothbrushing techniques, individualized dietary counseling, and systematic referral for regular dental check-ups. Interdisciplinary collaboration between pediatric dentists and diabetologists is essential to ensure early detection, prevention, and management of oral conditions in this high-risk population (29).

These findings suggest that preventive strategies in children with T1DM should prioritize the quality of oral hygiene practices over frequency alone, particularly through targeted behavioral interventions.

### 4.1 Limitations

These findings should be interpreted in light of certain limitations.

The control group was recruited from children participating in a national dental prevention program. Consequently, these children may have had greater exposure to oral health education and preventive care than participants with T1DM, potentially influencing oral hygiene behaviors and oral health outcomes. This should be considered when interpreting comparisons between groups. In addition, participants were not matched according to socioeconomic characteristics. Although parental educational level was recorded, other socioeconomic indicators were not collected. Therefore, the potential relationship between socioeconomic factors and oral health outcomes cannot be completely excluded.

A formal sample size calculation was not performed prior to recruitment. Consequently, the study may have been underpowered to detect small differences between groups. In addition, the convenience sampling method may have affected the generalizability of the findings. Nevertheless, the study is strengthened by its comparative design and the inclusion of a relatively large sample of children with T1DM.

Furthermore, only univariate analyses were performed. Consequently, potential confounding factors such as age, sex, parental education, oral hygiene practices, and dietary behaviors could not be simultaneously controlled for. Future studies incorporating multivariable regression models are warranted to identify independent predictors of oral health outcomes in children with T1DM.

The broad age range included in the study (3–17 years) may have introduced heterogeneity related to dentition stage, oral hygiene skills, dietary autonomy, and oral health status. Although participants were descriptively categorized according to age groups corresponding to primary, mixed, and permanent dentition stages, stratified analyses were not performed. Therefore, developmental differences may be linked to the observed outcomes and should be considered when interpreting the results. Dietary assessment was based on self-reported information provided by participants and/or their parents. No validated nutritional assessment instrument was used, and quantitative sugar intake was not measured. Therefore, some degree of reporting bias cannot be excluded.

Several variables, including oral hygiene practices, dietary habits, dental attendance, and parental knowledge, were obtained through self-reported questionnaires. Consequently, the findings may have been affected by recall bias, social desirability bias, and potential misclassification of behaviors. These sources of information bias should be considered when interpreting the results.

Future longitudinal studies incorporating multivariate analyses are warranted to better elucidate causal relationships and identify independent predictors of oral health outcomes in children with T1DM.

## 5 Conclusion

Children with type 1 diabetes exhibit poorer periodontal and oral hygiene conditions compared with non-diabetic peers, particularly in terms of plaque accumulation, calculus deposition, and gingival inflammation, while dental caries experience appears comparable between groups.

Preventive determinants—especially the quality of oral hygiene practices and dietary behaviors—were significantly associated with oral health outcomes in this population, highlighting the interaction between metabolic and behavioral factors.

These findings support the integration of targeted oral health promotion strategies into routine pediatric diabetes care. Strengthening preventive approaches, including oral hygiene education, dietary guidance, and regular dental follow-up, may help support better oral health among children with T1DM.

## Data Availability

The original contributions presented in the study are included in the article/Supplementary Material, further inquiries can be directed to the corresponding author.
